# A human gut metaproteomic dataset from stool samples pretreated or not by differential centrifugation

**DOI:** 10.1016/j.dib.2015.07.014

**Published:** 2015-07-26

**Authors:** Alessandro Tanca, Antonio Palomba, Salvatore Pisanu, Maria Filippa Addis, Sergio Uzzau

**Affiliations:** a Porto Conte Ricerche, Tramariglio, Alghero, Italy; b Dipartimento di Scienze Biomediche, Università di Sassari, Sassari, Italy

**Keywords:** Differential centrifugation, Gut microbiome, Human gut, Metaproteomics, Sample preparation

## Abstract

We present a human gut metaproteomic dataset deposited in the ProteomeXchange Consortium via the PRIDE partner repository with the dataset identifier PXD001573. Ten aliquots of a single stool sample collected from a healthy human volunteer were either pretreated by differential centrifugation (DC; *N*=5) or not centrifuged (NC; *N*=5). Protein extracts were then processed by filter-aided sample preparation, single-run liquid chromatography and high-resolution mass spectrometry, and peptide identification was carried out using Sequest-HT as search engine within the Proteome Discoverer informatic platform. The dataset described here is also related to the research article entitled “Enrichment or depletion? The impact of stool pretreatment on metaproteomic characterization of the human gut microbiota” published in Proteomics (Tanca et al., 2015), [1].

**Specifications table**

**Table:** 

Subject area	Biology
More specific subject area	Proteomics
Type of data	1) Raw mass spectrometry data
	2) Processed mass spectrometry data
	3) Overall protein and peptide identification tables (excel files)
How data was acquired	LTQ-Orbitrap Velos mass spectrometer interfaced with an UltiMate 3000 RSLCnano LC system (Thermo Scientific)
Data format	1) Raw (raw mass spectrometry files)
	2) msf (Proteome Discoverer output files)
	3) xlsx (Excel tables)
Experimental factors	Stool differential centrifugation
Experimental features	1) Protein extraction (mechanical disruption in SDS-based buffer)
	2) Filter-aided sample preparation (FASP)
	3) LC–MS/MS analysis
Data source location	Tramariglio, Alghero (Sassari), Italy
Data accessibility	ProteomeXchange Consortium, dataset identifier PXD001573 http://proteomecentral.proteomexchange.org/cgi/GetDataset?ID=PXD001573

**Value of the data**

• first metaproteomic analysis of pretreated and non-pretreated stool samples,
• over 5800 and 15,000 non-redundant proteins and peptides identified in total, respectively,
• deep information about gut microbiota and host proteins in the same experiment.

## 1 Materials and methods

### 1.1 Stool sample

Human feces were provided by a healthy volunteer who gave consent to their use for research purposes, as described previously [1]. Feces were split into ten samples, five of which underwent differential centrifugation (indicated with letters A–E in the Excel table), whereas the remaining five were directly subjected to protein extraction (indicated with letters F–J in the Excel table).

### 1.2 Differential centrifugation

Stool samples were subjected to differential centrifugation to enrich for microbial cells, according to VerBerkmoes et al. [2] and Tanca et al. [3], with minor modifications. Briefly, samples were resuspended in PBS to a final volume of 50 ml, vortexed, shaken in a tube rotator for 45 min, and subjected to low-speed centrifugation at 500×*g* for 5 min aimed to eliminate particulate and insoluble material. The supernatants were then carefully transferred to a clean polyallomer centrifuge bottle (Beckman Coulter, Brea, CA, USA) and kept at 4 °C, whereas the pellets were suspended again in PBS. The entire procedure was repeated for a total of three rounds. Finally, the supernatants (one per round, three per sample) were centrifuged at 20,000×*g* for 15 min, and the obtained pellets were subjected to protein extraction as described below.

### 1.3 Protein extraction and digestion

Samples were resuspended by vortexing in extraction buffer (2% SDS, 100 mM DTT, 20 mM Tris–HCl pH 8.8), then heated and subjected to a combination of bead-beating and freeze-thawing steps as detailed elsewhere [3]. Protein extracts were subjected to on-filter reduction, alkylation, and trypsin digestion according to the filter-aided sample preparation (FASP) protocol [4], with slight modifications detailed elsewhere [5], using Amicon Ultra-0.5 centrifugal filter units with Ultracel-10 membrane (Millipore, Billerica, MA, USA).

### 1.4 LC-MS/MS analysis

LC–MS/MS analysis was carried out using an LTQ-Orbitrap Velos mass spectrometer (Thermo Scientific) interfaced with an UltiMate 3000 RSLCnano LC system (Thermo Scientific). The single-run 1D LC peptide separation was performed as previously described [3,6], loading 4 μg of peptide mixture per each sample, and the mass spectrometer was set up in a data dependent MS/MS mode, with Higher Energy Collision Dissociation as the fragmentation method, as illustrated elsewhere [5].

### 1.5 Data analysis

Peptide identification was performed using Proteome Discoverer (version 1.4.1; Thermo Scientific), with a workflow consisting of the following nodes (and respective parameters): Spectrum Selector for spectra pre-processing (precursor mass range: 350–5000 Da; S/N Threshold: 1.5), Sequest-HT as search engine (Protein Database: see below; Enzyme: Trypsin; Max. missed cleavage sites: 2; Peptide length range 5–50 amino acids; Max. Delta Cn: 0.05; Precursor mass tolerance: 10 ppm; Fragment mass tolerance: 0.02 Da; Static modification: cysteine carbamidomethylation; Dynamic modification: methionine oxidation), and Percolator for peptide validation (FDR<1% based on peptide q-value). Results were filtered in order to keep only rank 1 peptides, and protein grouping was allowed according to the maximum parsimony principle.

The protein database was generated based on taxonomic information following an iterative approach, as proposed in a recent paper from our group [7]. Specifically, a preliminary search was performed against the complete UniProtKB database (release 2013_12). Then, the peptide sequences identified in all the samples through the preliminary search were uploaded into the Unipept web application (v.2.4, http://unipept.ugent.be) [8] to carry out a taxonomic assignment based on the lowest common ancestor (LCA) approach. In keeping with this, sequences from 298 detected microbial genera (from Archaea, Bacteria and Fungi) retrieved from UniProtKB (release 2013_12) were appended to the *Homo sapiens* sequences retrieved from SwissProt (release 2013_12) in order to generate a customized “host-microbiome” database containing sequences from specific microbial taxa and the host (5,990,075 protein sequences in total). Furthermore, an additional search was carried out using a “food” database containing all UniProtKB sequences belonging to the 6 most abundant plant genera detected in the preliminary search (namely, *Arachis*, *Musa*, *Corylus*, *Theobroma*, *Glycine* and *Pisum*; 117,047 total protein sequences), and the results were merged to those obtained with the “host-microbiome” database.
